# Artificial-Intelligence-Based Imaging Analysis of Stem Cells: A Systematic Scoping Review

**DOI:** 10.3390/biology11101412

**Published:** 2022-09-28

**Authors:** Julien Issa, Mazen Abou Chaar, Bartosz Kempisty, Lukasz Gasiorowski, Raphael Olszewski, Paul Mozdziak, Marta Dyszkiewicz-Konwińska

**Affiliations:** 1 Department of Diagnostics, Poznań University of Medical Sciences, Bukowska 70, 60-812 Poznań, Poland; 2 Doctoral School, Poznań University of Medical Sciences, Bukowska 70, 60-812 Poznań, Poland; 3 Department of Anatomy, Poznan University of Medical Sciences, 60-701 Poznan, Poland; 4 Prestage Department of Poultry Sciences, North Carolina State University, Raleigh, NC 27695, USA; 5 Department of Histology and Embryology, Poznan University of Medical Sciences, 60-701 Poznan, Poland; 6 Department of Veterinary Surgery, Institute of Veterinary Medicine, Nicolaus Copernicus University in Torun, 87-100 Torun, Poland; 7 Department of Medical Simulation, Poznan University of Medical Sciences, 60-701 Poznan, Poland; 8 Department of Oral and Maxillofacial Surgery, Cliniques Univeristaires Saint-Luc, UCLouvain, 1200 Brussels, Belgium; 9 Physiology Graduate Program, North Carolina State University, Raleigh, NC 27695, USA

**Keywords:** artificial intelligence, stem cells, induced pluripotent stem cells, embryonic stem cells, adult stem cells, imaging

## Abstract

**Simple Summary:**

Lately, investigations of artificial intelligence as an assisting tool for analyzing and identifying stem cells have increased. In this systematic scoping review, we aimed to identify and map the available artificial-intelligence-based techniques for imaging analysis, the characterization of stem cell differentiation, and trans-differentiation pathways. After an extensive search for the literature following a structured methodology, we included 27 studies in our systematic scoping review that we extracted the relevant data from. Based on the results of the included studies, artificial intelligence has the potential to serve as an assisting tool in stem cell imaging. However, it is still considered relatively new and under maturation. The goal of our review is to guide and help researchers while planning for future investigations.

**Abstract:**

This systematic scoping review aims to map and identify the available artificial-intelligence-based techniques for imaging analysis, the characterization of stem cell differentiation, and trans-differentiation pathways. On the ninth of March 2022, data were collected from five electronic databases (PubMed, Medline, Web of Science, Cochrane, and Scopus) and manual citation searching; all data were gathered in Zotero 5.0. A total of 4422 articles were collected after deduplication; only twenty-seven studies were included in this systematic scoping review after a two-phase screening against inclusion criteria by two independent reviewers. The amount of research in this field is significantly increasing over the years. While the current state of artificial intelligence (AI) can tackle a multitude of medical problems, the consensus amongst researchers remains that AI still falls short in multiple ways that investigators should examine, ranging from the quality of images used in training sets and appropriate sample size, as well as the unexpected events that may occur which the algorithm cannot predict.

## 1. Introduction

The emergence of stem cell research began in the year 1961, which marked the discovery of bone multipotent stem cells [1]. After several decades, the human pluripotent stem cells were first used in the preclinical stages of research which included isolating cells, implementing their functions, identifying their roles, and applying animal trials (such as injecting human-derived cardiomyocytes in damaged rodent hearts and witnessing their improvements) [2,3]. In the past few years, a trend in stem cell research has exponentially increased as it passed to the clinical stages, where the advancement of technology has enabled such innovation to potentially transition into human clinical trials in the future [2,3]. Stem cells are unspecialized human cells capable of self-renewal through mitosis, eventually forming more cells. Such a division generates two types of cells in which the first differentiate into a specific type of cell, whereas the second sustains self-renewal ability [4]. Three categories make up the stem cell division types which consist of induced pluripotent stem cells (iPSCs), embryonic stem cells (ECSs), and adult stem cells (ACSs) [5]. Pluripotent stem cells (PSCs) are defined by their ability to differentiate into the three layers of germ cells (ectoderm, mesoderm, and endoderm). Both iPSCs and ESCs are considered to be PSCs due to their ability to differentiate into the three germ layer derivatives, but a distinguishing feature between iPSCs and ESCs is that IPSCs are special reprogrammed somatic cells, generating pluripotent patient-specific cell lineages capable of aiding model human diseases [6]. Unlike iPSCs and ECSs, ACSs have a lower differentiation level, termed multipotent, and hence can differentiate into more tissue-specific stem cells [7]. ACSs are rare undifferentiated cells that spread throughout the entire body and transform into a proliferative state from their quiescent one in order to divide into new cells that would replace the naturally dying ones [7]. 

The future of stem cell-based therapy is becoming the precedent in advanced medicine. The potential for stem cell implementation grows with every experiment, bringing a new look at the possibilities of transplantology and regenerative medicine [8]. These therapies have targeted multiple medically severe conditions underscored by defective cell division or differentiation, such as cancer or congenital disabilities [8]. A wide variety of diseases are under the scope of stem-cell-based therapy in various fields of medicine, including cardiology (heart failure) [9] and ophthalmology (retinal and macular degeneration) [10]. Stem cells transformed the idea of treating what was once considered untreatable, for example certain neurodegeneration diseases, including Alzheimer’s and Parkinson’s [11]. In addition, arthroplasty has seen the impact of stem cells in various forms, such as healing tendon injuries, as well as in the cases of osteoarthritis, highlighting stem cell use in cartilage repair [12,13]. Stem cell research has also made its way to fertility disease, where the ability to produce sperm cells from iPSCs proved not only to be successful but also produced healthy and fertile mice [14,15]. Another field of medicine that has the potential to become a revolutionary step in stem cell research is diabetes, where induced stem cells differentiate into the missing pancreatic beta cells instead of transplanting them for a donor [16]. The most significant advancements are highlighted in hematopoietic stem cell research, earning them the title of being the most popular stem cell due to extensive experimentation and studies over the last fifty years, laying numerous foundations that have guided other medical fields in stem cell research and development [8]. As time progresses, research on stem cells is expanding beyond fields in medicine, reaching disciplines of dentistry and pharmacology.

The field of pharmacology has also been infiltrated by stem cell research. Notably, human-induced pluripotent stem cells (hiPSCs) saw their upbringing in 2009 when they were screened to model a type of neuropathic disease called familial dysautonomia, where multiple model features were discovered, and drugs targeting these features were manufactured and later tested [17]. Other examples include screening an anticancer drug, Bosutinib, which inhibits Src/c-Abl receptors, on hiPSCs extracted from amyotrophic lateral sclerosis (ALS) patients [18]. Now, the state of pharmacologic stem cell research has advanced tremendously, reaching the forefront of gene editing, CRISPR/cas9, and when combined with the stem cell application, can provide revolutionary input to the development of drug therapies that occurs through the integration of genes into hiPSCs, ultimately leading to both the development of therapeutic drug candidates and also the selection of the best drug out of these candidates [18]. Hence, such an approach to stem cells allows for systemic compound and drug evaluation regarding their safety, tolerability, and efficacy when applied for certain severe diseases due to drug screening on iPSCs [19].

The revolutionary advancements in technology, underlined by artificial intelligence (AI), have made stem cells available in terms of selecting the most suitable medication, establishing a diagnosis, and formulating risks and benefits when it comes to therapy [20]. In particular, emerging techniques of machine learning, deep learning, and convolutional neural networks (CNN) have assisted the framework of the reliable detection for various functions, including iPSC colony classifications [2], non-invasive cell therapy characterizations of normal versus abnormal cells [2], and image-based cellular morphology [2]. The accessibility of a wide variety of medical images combined with the continuously developing technology in the field of AI will take medicine to a whole different level [2]. The goal of the current research is to map and identify the available AI-based techniques for the imaging analysis of stem cells, the characterization of stem cell differentiation, and trans-differentiation pathways.

## 2. Materials and Methods

Guided by the Joanna Briggs Institute (JBI) methodology for a scoping review [21] and PRISMA-ScR (Transparent Reporting of Systematic Reviews and Meta-analyses Extension for Scoping Reviews) checklist [22], this systematic scoping review was conducted, as defined by its protocol that was developed previously by the research team [23]. 

### 2.1. Identifying the Research Question

Following the identification of the population, concept, and context (PCC) [21] components, a research question was developed. What are the available applications of AI-based imaging analysis for various types of stem cells?

The PCC component is established as follows:

- Population: stem cells;
- Concept: AI-based technique;
- Context: imaging analysis.

### 2.2. Searching Strategy

On 9 March 2022, we searched five electronic databases (PubMed, Medline, Web of Science, Cochrane, and Scopus) to identify inclusive studies based on a research strategy answering the research question (Table S1). Following a primary search, the complete searching strategy was then developed and customized for each database using specific queries. Additionally, we carried out a manual citation search of the retrieved data by screening the reference section of all studies to look for potential studies answering our research question.

The keywords included in the queries are as follows: algorithm, algorithm*, artificial intelligence, AI, automated, automatic, semi-automated, semi-automatic, deep learning, convolutional neural network, CNN, machine learning, stem cells, stem cell*, and imaging.

All the studies found were imported and stored in the Zotero 5.0 (Corporation for Digital Scholarship, Vienna, VA, USA) library. The library was deduplicated using web-based software and an SR accelerator [24], and a manual review by J.I was performed to confirm the removal of duplicates.

### 2.3. Eligibility Criteria

We generated the inclusion and exclusion criteria based on the PCC mnemonics [21].

Quantitative studies testing AI-based imaging analysis on any type of stem cell (iPSCs, ECSs, ACSs, and PSCs) of animals or humans were included without any language or date restrictions due to the novelty of this field. We excluded preprints and conference papers, as well as qualitative studies and quantitative studies investigating the use of AI technology for other purposes, including the imaging analysis of any type of cells rather than of stem cells. The studies where the full text was inaccessible were excluded. The inclusion and exclusion criteria are presented in Table 1.

### 2.4. Study Selection and Data Extraction

In the first phase of study selection, the title and abstract of all studies after deduplication were screened against the inclusion criteria by two independent reviewers (J.I. and M.A.C.). The reviewers met several times during this process; the first session was based on testing the study selection method and ensuring the understanding of the inclusion criteria by both reviewers. During the second phase, the reviewers again independently assessed the full text of the held studies following the first screening phase based on inclusion criteria. In case of any disagreements between reviewers at any stage, the opinion of the third reviewer (M.D.-K.) was taken, or the conflict was solved by discussion between the two reviewers. 

Data were extracted from the included studies by one reviewer (M.A.C.) and evaluated independently by the second reviewer (J.I.), and any disagreement between them was resolved by discussion or by the opinion of the third review (M.D.-K.). The data extraction tools were developed based on the JBI methodology for scoping review [21].

## 3. Results

### 3.1. Search Result

**Figure 1:**
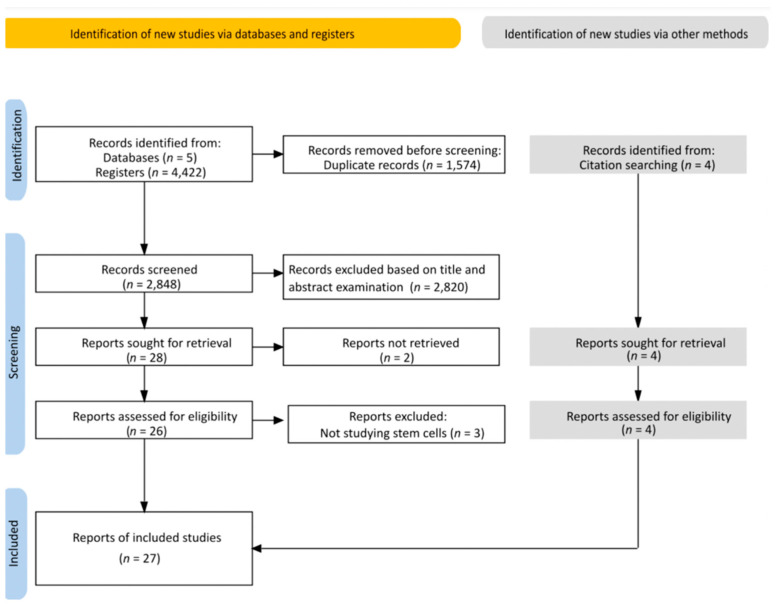
PRISMA flow diagram.

In total, 4422 articles were collected from the five electronic databases. Subsequently, 1574 articles were eliminated after deduplication of the library, progressing our screening for titles and abstracts against the inclusion criteria of 2848 studies. After the first screening phase, 28 articles were eligible for full-text analysis. While collecting the full text, two studies were eliminated due to the inaccessibility of the full text, and three other studies were excluded after the full-text screening as they were not studying stem cells. Finally, four additional articles were added through manual citation searching. In total, 27 studies were included in this systematic review (Figure 1). The level of the reviewer agreement was calculated using kappa statistics, K = 0.862, indicating a significant agreement between both reviewers.

### 3.2. Extracted Data

**Figure 2:**
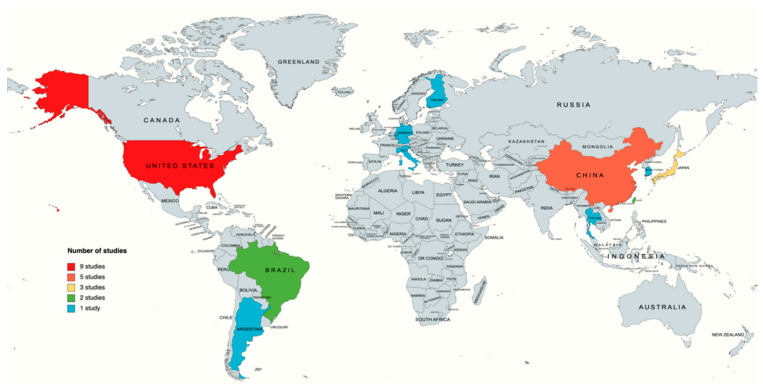
Geographic distribution of the retrieved publication studying stem cell image analysis using artificial intelligence.

**Figure 3:**
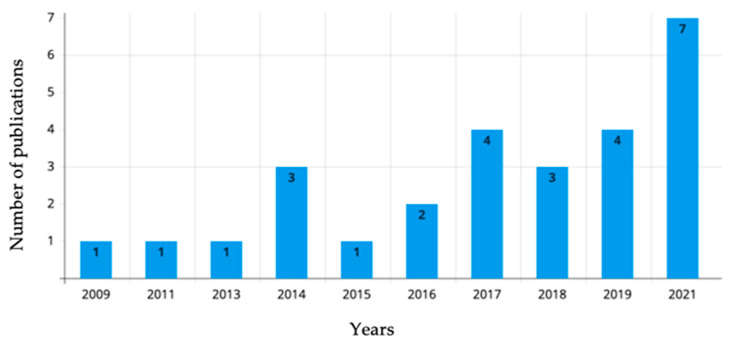
Bar chart showing the number of publications per year.

The extracted data will include the study author(s), the year of publication, the study location, the study aim, the type of cell, the sample size, the used algorithm, and the findings (Table 2). Additionally, we performed a demographic analysis to visualize the distribution of included studies on the world map with the number of publications per country (Figure 2) and a bar chart of the number of publications per year (Figure 3).

Upon a demographical analysis of the collected data, the United States was the highest contributing country in this field with 9 studies out of 27 (33.3%) [25,26,29,30,38,45,46,47,48], followed by China (13.5%) [27,32,41,42], Japan (11.1%) [28,33,37], and Brazil [50,51] and Taiwan [32,40] (both 7.4%). Argentina [34], Finland [43], Germany [39], Italy [49], South Korea [36], and Thailand [44] each had one study each (3.7%), as shown in Figure 2.

The number of publications studying stem cell image analysis using artificial intelligence increased throughout the years. Based on the included publications, the majority of published studies were in 2021 (seven studies) [25,26,27,28,29,30,31].

## 4. Discussion

A microscopic evaluation of all types of cultured cells is a routinely performed task in the laboratory setting. The contrast microscope is commonly used at various magnifications for cell analysis. The general criteria for cultured cells are confluence, dead cells, or severely atypical morphology. Therefore, operators must be trained in cell morphology and their ongoing relationship in all cases. The advanced approach to this task uses automatically extracted data that should include the most important parameters, including the cells’ confluence, cell-free areas, dead cells, and cell morphology changes. When performed manually, it suffers from low reproducibility and, on top of that, must be assessed quickly to ensure sufficient throughput. Some of the available software requires an invasive approach using either reporter genes or immunofluorescence labeling, which may lead to the irreversible modification of the cell sample or cell line death. Both systems require either cell modifications or cell sample sacrifice. Only methods that are non-invasive and capable of processing large extensive image data within a short time frame are applicable. They also need to be able to perform multi-class segmentation to assess all required parameters. Deep-learning-based algorithms are at the forefront of such complex tasks. 

This systematic scoping review aimed to identify the currently available AI methods which support the laboratory evaluation of stem cells. An analysis of the included studies revealed that the visual assessment of stem cells was mainly based on the morphological features of cells in colonies and the attempt to indicate their expected features. The potential of several algorithms was tested by authors to assess the level of differentiation and morphological changes during stem cell culture and an attempt to distinguish between different types of cells during their development stages based on the morphological features. An assessment made with the help of algorithms has a chance to be more effective and also less time-consuming. The key to developing successful algorithms is the correct selection of images, the use of appropriate data sets, and enough training images. Some of the studies described above boast a large sample size for training purposes; this significantly increases the reliability of the results.

### 4.1. iPSC

iPSCs have the potential to differentiate into all cell types, except extraembryonic cells, and possess a high proliferative capacity, and can be cultured on an industrial scale. These features make them an area of great interest for potential application in advanced therapies.

In the following section, we present the 16 studies that test the power of artificial intelligence in analyzing, identifying, and classifying human or animal iPSC.

Fischbacher et al. [25] tested the power of three algorithms (Monoqlo, RetinaNet, and ResNet) in the automatic detection of colony presence and the identification of clonality on approximately 30,000 images. Developed using the PerkinElmer HCS system, Guo et al. [27] tested the ability of the algorithm on high-content analysis to study embryo-like structures derived from several mice-induced PSC lines, TSCs and ESCs. Imamura et al. [28] built an ALS prediction model using a CNN-based deep learning algorithm where 4500, 1350, and 900 images were used for training, validation, and testing, respectively. Joy et al. [29] trained five different neural networks (FCRN-A, FCRN-B, U-Net, Residual U-Net, and Count-ception) to localize each cell nucleus individually in a human-induced pluripotent stem cell (hiPSC) colony, generating longitudinal measures of cell and cellular neighborhood properties.

Chang et al. [32] and Chang et al. [40] tested CNN and human iPSCs. Chang et al. [32] tested CNN’s ability to trace human iPS cell formation from CD34+ cord blood cells on 144 images. In contrast, Chang et al. [40] applied the CNN on 132 images for the automatic detection and localization of human iPSC regions in brightfield microscopy images.

Orita et al. [33] trained VGG16 using bright-field images of cultured human-induced pluripotent stem cell-derived cardiomyocytes (hiPSC-CMs). The sample size was divided into 14,000 images for training, 2000 for validation, and 2000 for testing.

Zhang et al. [35] used XGBoost to model an algorithm for iPS cell identification against MEFs in the same stage by using live-cell images during the early stages of iPSC reprogramming

Kavitha et al. [36] used a set of 169 phase-contrast microscopic images of iPSC colonies and five different machine learning algorithms (support vector machine (SVM), random forest (RF), multilayer perceptron (MLP), decision tree (DT), and adaptive boosting (Adaboost) classifier models) to evaluate the selected iPSC colony features (shape, statistics, spectrum, etc.) and to eventually characterize stem cells where SVM, RF, and Adaboost were significant outperformers amongst the algorithms used. With the utilization of LeNET and AlexNET, Kusumoto et al. [37] tested a morphology-based automatic method for identifying endothelial cells derived from iPSCs which required 640 images for training and 160 for validation. Buggenthin et al. [39] implemented RNNs and CNNs to evaluate how accurately a deep learning method can predict lineage choice in differentiating primary hematopoietic cells.

Fan et al. [41] utilized a modified version of AlexNET to quantitatively test the automatic and label-free classification and segmentation of iPSC colonies derived from humans and animals. Using the improved supervised normalized cut (ISNC) segmentation and k-means clustering algorithm, Li et al. [42] proposed a multi-stage framework system, CBMIA, which was tested on 81 microscopic human iPSC images.

Joutsijoki et al. [43] assessed the automated quality identification of iPSC colony images using multiclass support vector machines and scaled invariant feature transformation (SIFT), where feeder cells were included and not included, using 173 images.

Finally, two studies by Maddah et al. [47] and Maddah et al. [48] used recorded videos to test AI. Maddah et al. [47] successfully identified iPSC-derived cardiomyocytes in the analyzed cell culture based on an algorithm that captures the beating signals, implementing a hierarchical clustering algorithm trained using 500 recorded videos. Utilizing more than 500 time-lapse sequence images of iPSCs, Maddah et al. [48] tested and presented a framework for automated analysis of phase-contrast images of iPSCs to capture and quantify morphological changes during colony growth.

### 4.2. ESC

There is a high demand for the precise and automatic quantification of ESC pluripotency in challenging to evaluate the environment of mixed colonies with undifferentiated and differentiated cells. Seven analyzed studies classified the human and animal ESC using a different algorithm, presented in the following.

Guan et al. [26] used 27,603 unlabeled grayscale images for fine-tuning and 3559 labeled ones for training the model, aiming to develop a deep learning method for hESC classification on a dataset of videos. Random network (RandNet) achieved a classification accuracy of 97.23 ± 0.94%. Waisman et al. [34] focused on designing an algorithm capable of distinguishing an early-differentiating cell from pluripotent cells by utilizing the ResNet50 and DenseNET architecture. In total, 1116 images under various differentiation-inducing conditions of mouse ESCs were used. 

Theagarajan et al. [38] proposed a system for classifying hESCs into six categories using the CNN approach alone or in combination with Triplet CNN, achieving more than 94% accuracy. A total of 784 realistic unlabeled images were used to train, validate, and test the CNN for classifying hESCs images in a hierarchical system, allowing for their classifications into six categories.

Lou et al. [46] also addressed the segmentation issue, focusing mainly on nuclear segmentation by procuring MINS, which was developed on a MATLAB/C++-based segmentation tool on murine extraembryonic endoderm stem cells and ESCs. Paduano et al. [49] applied a MATLAB script pack and an orientation matching algorithm to mESCs. The developed algorithm was trained using 57 images and was able to properly process images. Faustino et al. [50] tested a developed algorithm for counting and detecting fluorescence microscopy images, where a total of 234 images of murine embryonic stem cells were used. Using a database of 92 images, Faustino et al. [51] implemented Java language 6.0 and Clipse 3.2 (development tool E) to shed light on fluorescent microscopy image use in automated embryonic stem counting and cell detection.

### 4.3. Other Stem cells

The presented studies in this section analyzed and identified different types of stem cells. Two studies classified hBMSCs, and the others screened NSCs and MAPCs using AI.

In their study, Mota et al. [30] proposed an objective approach for automatically classifying mesenchymal stem cell (MSC) efficacy using a training dataset of 71 images and a dataset of 36 images for validation. Bajcsy et al. [45] focused on three-dimensional (3D) focal segmentation in their algorithm process, where they managed to construct six new 3D segmentation algorithms using over a thousand z-stacks (3D images). Zhu et al. [31] trained and tested Xception, ResNet, VGGNet, and Inception-v730 to recognize the features of differentiated NSCs via un-labelled brightfield single-cell images. The number of NSC images used for training and testing was 19,533 and 29,895, respectively. Wuttisarnwattana et al. [44] delved deeper into fluorescent imaging and focused on cryo-imaging, specifically creating fluorescent images of MAPCs, from which 700 images were taken.

## 5. Conclusions

CNN is used to address several tasks and issues in the medical field and is extensively studied to implement its potential into the domain of stem cell biology in the form of automatic identification of cell types, their features, and development stages with the use of microscopic images without the necessity for molecular labeling. Deep learning technology has significantly improved, and the accuracy of the CNN image classification task has vastly exceeded that of humans. The segmentation process assigns each pixel in the image to an object class, making the classification of the image on the pixel level achievable within the boundary area.

Automatic algorithms outperform human-labeling skills in multiple ways, but proper annotation is still required to achieve this level of effectiveness. AI is at the forefront of accelerated progress in biomedical research and will majorly influence each stage of stem cell studies, consequently impacting the transfer of its research results to clinical practice. The amount of research in this field is significantly increasing with time. However, there are important factors affecting the final results that investigators should consider: the quality of images used as a training set, the sample size, and the elimination of unexpected events that the algorithm cannot predict.

## Figures and Tables

**Table 1:** Table of Inclusion and Exclusion Criteria.

Inclusion Criteria	Exclusion Criteria
Studies involving any type of Stem cells (iPSCs, ECSs, ACSs, PSCs)	Studies investigating different types of cells rather than stem cells
Studies using AI-based imaging analysis	Studies using AI technology for other purposes than imaging analysis
Published studies in any language	Reviews
No date restriction	Preprints and conference papers
Full-text accessible	Full-text not accessible

**Table 2:** Data extracted from included studies.

Author, Study, Location & Year of Publication	Study Aim	Cell Type	Sample Size	Algorithm	Findings
Fischbacher et al., USA, 2021. [25]	Automatic detection and identification of colony presence and clonality	Human-induced pluripotent stem cells (hiPSCs)	Approximately 30,000 images	Monoqlo RetinaNet ResNet	The algorithm was capable of analyzing the data volumes in less than an hour.
Guan et al., USA, 2021. [26]	Developing a deep learning classifiers for the classification of human embryonic stem cells (hESCs) on a video dataset	hESCs	27,603 unlabeled grayscale images and 3559 labeled grayscale images	Random network (RandNet)	The proposed approach achieved a classification accuracy of 97.23 ± 0.94%.
Guo et al., China, 2021. [27]	Setting up a workflow to use machine-learning-assisted high-content analysis to study embryo-like structures	Mouse iPSCs, ESCs, and trophectoderm stem cells (TSCs)	N/A	Algorithms developed by the PerkinElmer HCS system	The workflow was able to establish a robust, unbiased, and automated machine learning-based protocols.
Imamura et al., Japan, 2021. [28]	iPSC detection using deep learning for amyotrophic lateral sclerosis prediction	iPSCs	4500 images for training 1350 images for validation 900 images for testing	CNNs	The algorithm achived an average accuracy of 0.90 ± 0.10 for cell classification.
Joy et al., USA, 2021. [29]	Training a group of neural networks to localize individual cell nucleus in an hiPSC colony, and to generate longitudinal measures of cell and cellular neighborhood properties	hiPSCs	12 time lapse movies	FCRN-A FCRN-B U-Net Residual U-Net Count-ception	The trained group of neural networks was able to identify the characteristics of multicellular organization at the single-cell local neighborhood and whole-colony scales.
Mota et al., USA, 2021. [30]	Proposing an objective aproach that determines the morphological phenotypes of mesenchymal stem cells (MSCs) for culture efficacy prediction	Human bone-marrow-derived MSCs (hBMSCs)	Training dataset 71 images Validation dataset 36 images	Proposed a new algorithm generated using MATLAB	The proposed method showed 88% sensitivity and 86% precision for overall cell detection.
Zhu et al., China, 2021. [31]	Building a CNN system that uses unlabelled brightfield single-cell images to recognize differentiated neural stem cell (NSC) features	NSCs	119,533 images for training 29,895 images for testing	Xception ResNet VGGNet Inception-v730	The model estimated the proportion of final cell-type differentiation in early stages of differentiation before the common laboratory techniques were able to detect it.
Chang et al., Taiwan, 2019. [32]	Establishing a traceable method for human iPSC formation from CD34+ cord blood cells	CD34+ cells	144 images	CNNs	The machine learning method provided a time-series visualization and quantitative analysis of the hiPSC induction and transition process.
Orita et al., Japan, 2019. [33]	Training a CNN model using bright-field images	hiPSC-derived cardiomyocytes (hiPSC-CMs)	14,000 images for training 2000 images for validation 2000 images for testing	VGG16	The tested model showed an average of 0.897 ± 0.01 accuracy, 0.946 ± 0.005 precision, 0.843 ± 0.02 recall, and 0.890 ± 0.01 F1-score.
Waisman et al., Argentina, 2019. [34]	Training CNN to distinguish the pluripotent stem cells from early-differentiating cells based on cellular morphology	Mouse embryonic stem cells (mouse ESCs)	1116 images	ResNET50 DenseNet	The tested model was able achieve distinguishment with a 99% accuracy.
Zhang et al., China, 2019. [35]	Proposing a machine-learning-based approach to detect iPS progenitor cells during the early stage of reprogramming and against normal mouse embryonic fibroblasts (MEFs) in the same stage	iPS progenitor cells and MEFs	N/A	XGBoost	The model predicted iPS progenitor cells with a minimum precision of 52% and a maximum precision of 75%.
Kavitha et al., South Korea, 2018. [36]	Evaluating several machine learning classifiers for iPSC colony characterization based on a quantitative texture extraction	iPSC and inactive MEFs	169 phase-contrast microscopic images	Support vector machine (SVM) Random forest (RF) Multilayer perceptron (MLP) Decision tree (DT) Adaptive boosting (Adaboost) classifier models	SVM, RF, and Adaboost delivered better classification performances than DT and MLP. The proposed automated fused statistical, shape-based, and moment-based texture pattern features that are potentially more helpful to biologists for characterizing the colonies of stem cells.
Kusumoto et al., Japan, 2018. [37]	Testing an automated method for identifiying iPSC-derived endothelial cells based on morphology		640 images for training 160 images for validation 600 images for testing	LeNet AlexNet	The deep learning technique was able to detect iPSC-derived endothelial cells with 90% accuracy.
Theagarajan et al., USA, 2018. [38]	Proposing a system for hESC images image classification using CNN and triplet CNN in a hierarchical system which allows for their classifications into 6 categories	hESCs	784 images	Conv Maxpool FC Layer	The proposed system classified hESC images with 85.67% accuracy using the CNN Alone and recorded a 91.38% accuracy using the CNN and Triplet CNN and 94.11% accuracy by fusing the outputs of the CNN and triplet CNNs.
Buggenthin et al., Germany, 2017. [39]	Testing a deep learning method that predicts the lineage choice in the differentiating primary hematopoietic progenitors	Murine hematopoietic stem and progenitor cells (HSPCs)	2,400,000 image patches	CNN Recurrent neural network (RNN)	Without a molecular labeling, the algorithm was able to identify cells with differentially expressed lineage-specifying genes.
Chang et al., Taiwan, 2017. [40]	Automatic detection and localization of human iPSC regions in brightfield microscopy images	CD34+ cells	132 images	CNN	The automatic method successfully localized and detected human iPS cell formation, ultimately producing an iPS cell culture perk.
Fan et al., China, 2017. [41]	Testing a label-free and quantitative automated system for iPSCs segmentation and classification	iPSCs (human and animals)	50 images	Modified AlexNET	No significant differences were recorded between the used algorithm and the manual method for cell classification.
Li et al., China, 2017. [42]	Proposing a system of multi-stage frameworks using content-based microscopic image analysis (CBMIA)	hiPSCs	81 microscopic images	Improved supervised normalized cut (ISNC) segmentation algorithm k-means clustering algorithm	Results show that the CBMIA system was able to support a high-performing clustering result, allowing for the prediction of the stem cell differentiation process.
Joutsijoki et al., Finland, 2016. [43]	Assessing the automated quality of iPSC colony image identification where feeder cells are included and not included	iPSCs	173 images	Multiclass support vector machines Scaled invariant feature transformation (SIFT)	The k-NN classifier achieved accurate results with an accuracy of 62.4%.
Wuttisarnwattana P et al., Thailand, 2016. [44]	Describing a novel machine-learning-based approach for detecting fluorescently labeled stem cells in cryo-imaging data	Mouse multipotent adult progenitors cells (MAPCs)	700 fluorescent images	A novel algorithm created using MATLAB	The new tested software allowed for an accurate detection and quantification of cells anywhere in the entire whole mouse volume with single-cell sensitivity.
Bajcsy et al., USA, 2015. [45]	Designing algorithms that can be applied to a very large number of confocal microscopy images (z-stacks) for three-dimensional (3D) segmentation	hBMSCs	More than 1000 z-stacks	A set of six newly constructed 3D segmentation algorithms	The most accurate 3D segmentation algorithm achieved an average precision of 0.82 and accuracy of 0.84 measured by the Dice similarity index.
Lou et Al., USA, 2014. [46]	Developing a modular interactive nuclear segmentation (MINS) as a MATLAB/ C++-based segmentation tool tailored for counting cells and fluorescent-intensity measurements	Murine extraembryonic endoderm stem and embryonic stem cells (ESCs)	N/A	Seeded geodesic image segmentation (SGIS)	The framework achieved a balance between computational complexity and runtime.
Maddah et al., USA, 2014. [47]	Presenting a new method that can reliably extract and quantify beat signals from cardiomyocyte cell cultures	iPSC-derived cardiomyocytes	More than 500 videos	Hierarchical clustering algorithm	The use method was able to properly characterize stem-cell-derived cardiomyocytes.
Maddah et al., USA, 2014. [48]	Presenting a framework for the automated analysis of phase-contrast images of stem cells to capture and quantify morphological changes during colony growth	iPSCs	Over 500 time-lapse sequences images	N/A	The proposed novel framework demonstrated the successful classification of stem cells based on texture pattern recognition.
Paduano et al., Italy, 2013. [49]	Developing an analysis pipeline which can automatically process images of stem cell colonies in optical microscopy in order to study markers of embryonic stem cells (ESCs) heterogeneity	Mouse ESCs	57 images	A proposed approach in MATLAB, CLAHE (image adjustment) Orientation matching algorithm	The tested algorithm achieved proper image processing.
Faustino et al., Brazil, 2011. [50]	Presenting an algorithm that counts and detects ESCs in fluorescence microscopy images	Murine ESCs	234 images	Developed their own graph-mining algorithm	The used method achieved an average F-measure above 90%.
Faustino et al., Brazil, 2009. [51]	Proposing an automatic method for ESC detection and counting under fluorescence microscopy images	ESCs	92 images	The algorithm was implemented in Java language 6.0 using the development tool Eclipse 3.2	The used method resulted in an average of 93.97% precision, recall 92.04%, and 92.87% F-measure.

## Data Availability

The data that support the findings of this study are available from the corresponding author upon reasonable request.

## Supplementary Materials

The following supporting information can be downloaded at: https://www.mdpi.com/article/10.3390/biology11101412/s1. Table S1: Searching stagey.
