# Supplementary material for: Artificial-Intelligence-Based Imaging Analysis of Stem Cells: A Systematic Scoping Review
Source: Biology (Basel). 2022 Sep 28;11(10):1412. doi: 10.3390/biology11101412 (PMC9598508; doi:10.3390/biology11101412)
Supplement: Supplementary file 1 [file biology-11-01412-s001.zip › biology-1925359-supplementary.pdf]

Table S1. Searching stagey:

Artificial intelligence-based imaging analysis of stem cells: a systematic scoping review

Searched databases: PubMed, Medline, Web of Science, Cochrane (Wiley), and Scopus
Searching Date: 09/03/2022
No filters applied and no publication date restrictions.

1- PubMed:

Search	Query	Field	Records retrieved
#1	(algorithm) OR (algorithm*) OR ("artificial intelligence") OR (AI) OR (automatic) OR (automated) OR (semi-automatic) OR (semi-automated) OR ("deep learning") OR ("convolutional neural network") OR (CNN) OR ("machine learning")	All Field	1,837,309
#2	("stem cells") OR ("stem cell*")	All Field	448,711
#3	("imaging")	All Field	2,356,758
#4	#1 AND #2 AND #3		1,377

2- Medline (Ovid):

Search	Query	Field	Records retrieved
#1	algorithm OR algorithm* OR artificial intelligence OR AI OR automatic OR automated OR semi-automatic OR semi-automated OR deep learning OR convolutional neural network OR CNN OR machine learning	mp. [mp=title, abstract, original title, name of substance word, subject heading word, floating sub-heading	728,039

		word, keyword heading word, organism supplementary concept word, protocol supplementary concept word, rare disease supplementary concept word, unique identifier, synonyms]	
#2	stem cells OR stem cell*	mp. [mp=title, abstract, original title, name of substance word, subject heading word, floating sub-heading word, keyword heading word, organism supplementary	407,309

		concept word, protocol supplementary concept word, rare disease supplementary concept word, unique identifier, synonyms]	
#3	imaging	mp. [mp=title, abstract, original title, name of substance word, subject heading word, floating sub-heading word, keyword heading word, organism supplementary concept word, protocol supplementary concept word, rare	2,265,597

		disease supplementary concept word, unique identifier, synonyms]	
#3	#1 AND #2 AND #3		479

Cochrane (Wiley):

Search	Query	Field	Records retrieved
#1	algorithm OR algorithm* OR "artificial intelligence" OR AI OR automatic OR automated OR semi-automatic OR semi-automated OR deep learning OR "Convolutional neural network" OR CNN OR "machine learning"	Title/Abstract/Key words	39,068
#2	"stem cells" OR "stem cell*"	Title/Abstract/Key words	146,58
#3	"imaging"	Title/Abstract/Key words	802,77
#4	#1 AND #2 AND #3		29

Web of Science:

Search	Query	Field	Records retrieved
#1	ALL=(algorithm OR algorithm* OR "artificial intelligence" OR AI OR automatic OR automated OR semi-automatic OR semi-automated OR "deep learning" OR "Convolutional neural network" OR CNN OR "machine learning")	Indexes=SCI-EXPANDED, SSCI, A&HCI, CPCI-S, CPCI-	3,896,415

		SSH, BKCI-S, BKCI-SSH, ESCI, CCR-EXPANDED, IC Timespan=All	
#2	ALL=("stem cells" OR "stem cell*")	Indexes=SCI-EXPANDED, SSCI, A&HCI, CPCI-S, CPCI-SSH, BKCI-S, BKCI-SSH, ESCI, CCR-EXPANDED, IC Timespan=All	567,490
#3	ALL=(imaging)	Indexes=SCI-EXPANDED, SSCI, A&HCI, CPCI-	3,216,001

		S, CPCI-SSH, BKCI-S, BKCI-SSH, ESCI, CCR-EXPANDED, IC Timespan=All	
#4	#1 AND #2 AND #3		1,618

Scopus:

Search	Query	Field	Records retrieved
#1	TITLE-ABS-KEY (algorithm OR algorithm* OR "artificial intelligence" OR ai OR automatic OR automated OR semi-automatic OR semi-automated OR "deep learning" OR "Convolutional neural network" OR cnn OR “machine learning”)	Article title, Abstract, Keywords	4,859,094
#2	TITLE-ABS-KEY ("stem cells" OR "stem cell*")	Article title, Abstract, Keywords	531,876
#3	TITLE-ABS-KEY (imaging)	Article title, Abstract, Keywords	2,623,179
#3	#1 AND #2 AND #4		919
